# A Rationale for the Use of Ivabradine in the Perioperative Phase of Cardiac Surgery: A Review

**DOI:** 10.3390/jcdd12080294

**Published:** 2025-07-31

**Authors:** Christos E. Ballas, Christos S. Katsouras, Konstantinos C. Siaravas, Ioannis Tzourtzos, Amalia I. Moula, Christos Alexiou

**Affiliations:** 1 Department of Cardiac Surgery, University Hospital of Ioannina, 45500 Ioannina, Greece; 2 First Department of Cardiology, University Hospital of Ioannina, 45500 Ioannina, Greece; cskats@yahoo.com (C.S.K.); siaravaskon@gmail.com (K.C.S.); 3 Second Department of Cardiology, University Hospital of Ioannina, 45500 Ioannina, Greece; ioannistzourtz@gmail.com; 4 Department of Surgery, “Achillopouleion” General Hospital, 38222 Volos, Greece; amaliamoula1@gmail.com

**Keywords:** hyperpolarization-activated cyclic nucleotide-gated channels, ivabradine, cardiac surgery

## Abstract

This review explores the advantages of ivabradine in the management of cardiac surgery patients, particularly highlighting its heart rate (HR)-reducing properties, its role in minimizing the impact of atrial fibrillation, and its contributions to improving left ventricular diastolic function, as well as reducing pain, stress, and anxiety. In parallel, studies provide evidence that ivabradine influences endothelial inflammatory responses through mechanisms such as biomechanical modulation. Unlike traditional beta-blockers that may induce hypotension, ivabradine selectively inhibits hyperpolarization-activated cyclic nucleotide-gated (HCN) channels, allowing for effective HR reduction without compromising blood pressure stability. This characteristic is particularly beneficial for patients at risk of atrial fibrillation post-surgery, where HR control is crucial for cardiovascular stability. This is an area in which ivabradine appears to play a role prophylactically, possibly in combination with beta-blockers. Furthermore, ivabradine has been associated with enhanced diastolic parameters in left ventricular function, reflecting its potential to improve surgical outcomes in patients with compromised heart function. In addition to its cardiovascular benefits, it appears to alleviate psychological stress and anxiety, common in postoperative settings, by moderating the neuroendocrine response to stress, thereby reducing stress-induced hormone levels. Furthermore, it has notable analgesic properties, contributing to pain management through its action on HCN channels in both the peripheral and central nervous systems. Collectively, these findings indicate that ivabradine may serve as a valuable therapeutic agent in the perioperative care of cardiac surgery patients, addressing both physiological and psychological challenges during recovery.

## 1. Introduction

Hyperpolarization-activated cyclic nucleotide-gated (HCN) channels, encompassing the HCN 1–4 isoforms, play integral roles in both the heart and central nervous system. In the cardiac context, HCN channels are pivotal for pacemaker activity in the sinoatrial node, with the If current contributing significantly to spontaneous diastolic depolarization and HR regulation, influenced by sympathetic and parasympathetic stimulation through cyclic adenosine monophosphate (cAMP) modulation [1,2]. Conversely, in the nervous system, HCN channels facilitate critical processes such as action potential generation, synaptic plasticity, and network oscillations, underscoring their significance in modulating neuronal excitability [3,4,5].

The differential expression of HCN channel isoforms across cardiac and neuronal tissues contributes to their functional diversity, particularly in response to hyperpolarization and cyclic nucleotide signaling [1,6]. This unique gating mechanism distinguishes them from typical voltage-gated ion channels, highlighting their relevance as therapeutic targets in various pathophysiological conditions, including arrhythmias and neurophysiological states [2,3,5].

Research has suggested that ivabradine, which inhibits the pacemaker If current in the sinoatrial node, mediated by the HCN 4 channel, may provide a unique advantage in the cardiac surgery context by lowering HR without negatively impacting myocardial contractility or inducing significant bradycardia. This specific characteristic makes ivabradine an attractive alternative for patients for whom beta-blockers pose a risk due to conduction abnormalities [7].

As the surgical population continues to evolve in complexity, understanding the implications of medication choice in the perioperative phase becomes increasingly important. Investigations have begun comparing the efficacy of ivabradine against beta-blockers, suggesting potential benefits of integrating ivabradine into postoperative protocols, particularly for those with left ventricular dysfunction. Studies show some promising outcomes when ivabradine is used in conjunction with beta-blockers. However, consistency in results varies, highlighting the necessity for a comprehensive evaluation of ivabradine’s effects on both cardiac function and rhythm stability in the diverse postoperative population of cardiac surgery patients [8].

Evidence synthesized from preclinical and clinical studies indicates that ivabradine has multifaceted pharmacological effects that extend well beyond the reduction in HR. Its analgesic and anti-inflammatory actions, mediated through the inhibition of HCN channels in peripheral sensory neurons, open potential avenues for pain management. Furthermore, its ability to modulate neuroendocrine stress responses and improve cognitive and behavioral outcomes, as well as its favorable impact on diastolic function and potential reduction in postoperative atrial fibrillation (AF), highlight additional therapeutic roles in both the neurological and cardiovascular domains [7,9,10,11].

This review seeks to synthesize the current literature surrounding ivabradine’s role in cardiac surgery patients, focusing on its effectiveness in reducing atrial fibrillation rates, enhancing hemodynamic stability, reducing inflammatory response after surgery, reducing levels of stress, pain, and anxiety, and minimizing adverse events related to conventional beta-blocker therapies.

## 2. Pathophysiological Effects of Ivabradine

### 2.1. Heart Rate Control

HCN channels play crucial roles across various physiological systems, including the heart and central and peripheral nervous systems. In the heart, particularly the HCN 4 subunit, HCN channels are indispensable for pacemaker activity, generating the “funny current” (If) that regulates heart rhythm. Ivabradine is a selective inhibitor of the HCN channel, specifically targeting the If current in the sinoatrial node. This mechanism inhibits the channel’s function, thereby stabilizing the diastolic depolarization slope and resulting in a reduced HR in a dose-dependent manner, without affecting conduction times within the atrioventricular node. As a result, ivabradine is noted for its ability to lower the HR while preserving ventricular contractility without adverse blood pressure effects [8,12].

Studies have indicated that ivabradine may contribute to improved hemodynamic stability in patients with inappropriate sinus tachycardia, especially during the perioperative period of CABG or after heart transplantation [12,13]. Bhatt et al. conducted a clinical study that included 150 patients who developed inappropriate sinus tachycardia after coronary artery bypass grafting (CABG) operation. The patients were randomized into three treatment groups. The first group received metoprolol, the second one received ivabradine, and the last one received a combination of both. A decrease in HR was observed in all patients, but this reduction was significant (*p* < 0.05) in the combination group, followed by the ivabradine group, which was significantly greater than the metoprolol-only treated group [12]. Heart transplant recipients may present with increased left ventricular mass due to the chronically elevated HR, which is attributed to the denervation. Ivabradine’s mechanism of action allows for a selective reduction in HR without impairing myocardial contractility, thus preserving cardiac output even when heart rate is reduced. The findings reported by Zhang et al. indicate a significant reduction in heart transplant-related tachycardia sustained over an extensive follow-up period of 48 months, supporting that ivabradine is both effective and well-tolerated by this patient population. Furthermore, the implications of ivabradine use extend beyond mere HR modulation. By effectively managing tachycardia, ivabradine also contributes to left ventricular remodeling, as demonstrated in Doesch et al.’s study, where a considerable reduction in left ventricular mass was observed alongside prolonged HR reduction using ivabradine. This suggests that controlling HR post-transplant not only alleviates symptoms of tachycardia but may also help mitigate long-term structural changes in the heart, ultimately influencing the long-term prognosis of transplant recipients [14,15].

Unlike most beta-blockers, which can induce hypotension alongside bradycardia, ivabradine’s mechanism allows for a more favorable hemodynamic profile without significant decreases in blood pressure, which is critical in managing patients with cardiovascular instability post-surgery, where operative trauma, anesthesia effects, and fluid shifts exacerbate cardiac stress [16,17].

### 2.2. Inflammation and Oxidative Stress

Ivabradine has notable vascular benefits due to its effects on endothelial function. Recent studies suggest that ivabradine, by reducing HR, promotes shear stress-dependent anti-inflammatory mechanisms in arteries. This has been demonstrated in both in vitro and in vivo studies, where ivabradine influenced endothelial cell behavior, reducing inflammation and oxidative stress (Table 1).

The protective effects observed are mediated through pathways involving nitric oxide and related signaling proteins such as endothelial nitric oxide synthase (eNOS) and mammalian target of rapamycin (mTOR), which help mitigate vascular inflammation and oxidative stress. eNOS is a crucial enzyme in maintaining endothelial activity, while mTOR regulates protein synthesis, cell growth and proliferation, autophagy, cell metabolism, and stress responses. Ivabradine affects the pathways that involve these enzymes, preventing wall shear stress-induced endothelial inflammation independently of HR reduction [18]. An animal study showed that treatment with ivabradine reduced the expression of pro-inflammatory cytokine vascular cell adhesion molecule 1 (VCAM-1) and enhanced the expression of eNOS [19]. On the contrary, Custodis et al. showed that ivabradine-induced HR reduction in apolipoprotein E-deficient mice did not influence eNOS expression but decreased monocyte chemotactic protein-1 (MCP-1) mRNA and exerted potent antioxidative effects. Furthermore, it reduced vascular nicotinamide adenine dinucleotide phosphate (NADPH) oxidase activity and decreased markers of superoxide production and lipid peroxidation in the aortic wall, promoting better endothelial function [20]. In another animal study, ivabradine improved endothelial function, which was associated with decreased vascular reactive oxygen species (ROS) production due to reduced NADPH oxidase activity and the prevention of eNOS uncoupling [21].

### 2.3. Left Ventricular Diastolic Function

The role of ivabradine extends to the management of left ventricular (LV) diastolic dysfunction, a common complication subsequent to CABG. Research has elucidated that treatment with ivabradine may lead to significant improvements in diastolic parameters, thereby enhancing overall cardiac function post-surgery.

Cacciapuoti et al. evaluated 25 patients with “diastolic heart failure”, with a mean LV ejection fraction (LVEF) = 48 ± 0.20, who belonged to the New York Heart Association (NYHA) functional class II–III (in sinus rhythm). In these patients, ivabradine per os (5 mg/twice a day) was added to the conventional medical therapy (mainly b-blockers, angiotensin-converting enzyme inhibitors, and angiotensin-receptor blockers) and given for 12 weeks. They showed that there was an improvement in echocardiographic indices of LV diastolic function beyond the reduction in HR, concerning parameters of diastolic mitral inflow, pulmonary vein flow, and tissue Doppler imaging [22].

Panayotova et al. investigated the effect of ivabradine treatment in the early postoperative stages in patients who underwent a CABG operation and showed that many echocardiographic markers of diastolic LV dysfunction improved. These were the e’ wave, the ratio E/e’, the mitral flow deceleration time, and the left atrial volume [23]. In a recent prospective cohort study, which included sixty adult patients with coronary artery disease and preserved systolic LV function who underwent CABG surgery, the effect of adding ivabradine to standard postoperative therapy on indices of LV diastolic function was investigated. The authors also showed that ivabradine appears to significantly enhance echocardiographic parameters of diastolic dysfunction at 90 days following CABG operation [24].

## 3. The Role of Ivabradine Treatment in Specific Perioperative Clinical States

### 3.1. Postoperative Low Cardiac Output Syndrome (Table 2)

Several recent studies have illustrated the efficacy of ivabradine as an adjunct therapy in heart failure-associated low cardiac output syndromes. One study highlighted that ivabradine significantly reduces resting HR and improves LVEF, indicating enhanced cardiac function, which is critical in managing low cardiac output conditions [25]. Evidence indicates that ivabradine improves exercise tolerance and exerts favorable effects on myocardial remodeling, potentially reversing the pathological processes involved in heart failure and low cardiac output states [26,27].

Treatment with ivabradine could be beneficial in patients experiencing low cardiac output syndrome (LCOS) after cardiac surgery. In a phase 2 randomized controlled trial, patients with LVEF < 40% and sinus tachycardia following dobutamine infusion for LCOS after CABG operation received either intravenous ivabradine or placebo until dobutamine weaning or for up to 48 h. The authors showed that in the ivabradine treatment group, a significant control of HR in the target range of 80–90 bpm was observed, compared to the placebo group (*p*  < 0.001 vs. *p*  =  0.125, respectively), together with a significant increase in stroke volume (*p* < 0.001 vs. non-significant, respectively), systolic blood pressure (*p* < 0.05 vs. not significant, respectively), and cardiac output (*p* < 0.05 vs. not significant, respectively) [28].

In a recent retrospective study, which included 174 patients who underwent off-pump CABG surgery, intraoperative arrhythmias and hypotension were recorded after the patients were divided into two treatment groups [Group I (*n* = 90), who received ivabradine 5 mg/day b.i.d., and Group M (*n* = 84), who received metoprolol 50 mg/day b.i.d., five days before surgery until the postoperative day 10]. The mean HR was significantly lower during distal anastomosis in Group I than in Group M (*p* < 0.001), but two patients in each group developed low cardiac output [2 (2.22%) vs. 2 (2.38%), *p* = 1], who eventually died. The rates of hypotension and intraoperative inotropic support did not significantly differ between the two groups (*p* = 0.47 and *p* = 0.87, respectively). Indeed, it should be emphasized that the method for documenting the state of low cardiac output is not adequately described, and important hemodynamic data such as postoperative ejection fraction, stroke volume, and markers of left ventricular diastolic function have not been recorded. Therefore, this study does not provide reliable data regarding the effect of ivabradine on postoperative cardiac output [29].

**Table 2:** Studies examining the role of ivabradine in postoperative LCOS. LCOS: low cardiac output syndrome, HF: heart failure, EF: ejection fraction, HR: heart rate, LVEF: left ventricular ejection fraction, CABG: coronary artery bypass graft, CI: cardiac index, SV: stroke volume, BP: blood pressure, and N/A: not applicable.

Study (Year)	Type of Study	Sample Size	Design of the Study	Clinical Effect	Possible Role in a Specific Perioperative Clinical Context
Shah et al. (2024) [25]	Prospective clinical trial	200 patients	Patients admitted with acute HF were randomized to receive either standard HF treatment alone or standard treatment with adjunctive ivabradine therapy	The ivabradine treatment group had a significantly greater increase in EF (*p* < 0.001) and HR reduction (*p* < 0.05)	Postoperative LCOS
Nguyen et al. 2018 [28]	Phase 2, multicenter, single-blind, and randomized controlled trial	19 patients	Patients with LVEF < 40% presenting sinus tachycardia following dobutamine infusion for LCOS after CABG operation received either ivabradine or placebo	Ivabradine decreased HR (*p* < 0.001) and increased CI (*p* = 0.02), SV (*p* < 0.001), and systolic BP (*p* = 0.03)	Postoperative LCOS
Tekin et al. 2022 [29]	Single-center and retrospective study	174 patients	Patients who underwent off-pump CABG were divided into Group I, who received ivabradine, and Group M, who received metoprolol before surgery until postoperative day 10	The rates of hypotension and intraoperative inotropic support did not differ significantly (*p* = 0.47 and *p* = 0.87, respectively)	N/A

### 3.2. Postoperative Atrial Fibrillation

The emergence and management of postoperative atrial fibrillation (POAF) following cardiac surgery is a significant clinical challenge, likely through mechanisms of reducing the inflammatory response and its influence on diastolic cardiac function. Studies indicate that in the context of CABG, the use of ivabradine leads to significantly reduced rates of POAF when combined with traditional beta-blockade approaches [7,8,30] (Table 3). For example, one study demonstrated a marked reduction in AF incidence in patients receiving ivabradine alongside bisoprolol, with rates reported at 4% compared to 36% in control groups (*p* = 0.01). Furthermore, the inclusion of ivabradine was linked to decreased systolic and diastolic blood pressures and a reduction in intensive care unit (ICU) stay, suggesting not only efficacy in arrhythmia prevention but also overall improvement in postoperative recovery metrics [30].

A multicenter observational study enrolled 740 consecutive patients scheduled for elective CABG operation with or without valve surgery and examined the incidence of AF at 30-day follow-up, depending on perioperative treatment with ivabradine ± b-blocker. Patients were assigned to the following groups: ivabradine group, bisoprolol group, and combination treatment group. At the end of the 30-day follow-up, AF occurred in 77 patients. Ten patients (4.2%) in the third group developed AF, compared to 32 (15.1%) in the first group and 35 (12.2%) in the second group (*p* < 0.001). A statistically significant difference between the incidence of AF in group 1 and group 3 (*p* < 0.001) and between group 2 and group 3 (*p* = 0.008) was shown. No similar result was obtained when comparing groups 1 and 2 (*p* = 0.84) [7].

In the randomized clinical trial by Iliuță and Rac-Albu, 527 patients with conduction abnormalities or LV systolic dysfunction who underwent CABG or valvular surgery were randomized and given ivabradine, metoprolol, or metoprolol plus ivabradine. In-hospital POAF occurred less frequently with combined therapy than with metoprolol or ivabradine alone (*p* < 0.001). The associated relative risk showed a higher protective value for the occurrence of POAF in patients treated with combined double therapy compared to metoprolol monotherapy (−2.9 vs. −1.8). In addition, the rates of the study’s endpoints (30-day mortality, in-hospital AF/arrhythmias, in-hospital atrioventricular block/need for pacemaker, or in-hospital heart failure deterioration) were lower in the combined treatment group than the monotherapy groups (*p* < 0.0001). Finally, the researchers showed that the ivabradine treatment (as monotherapy or combined therapy) was superior compared to metoprolol treatment alone in terms of the aforementioned study’s endpoints, which include the incidence of POAF [8].

## 4. The Role of Ivabradine in the Modulation of Neural and Psychological Responses

### 4.1. HCN Channels and Neurotransmission

HCN channels facilitate important neuronal functions, such as rhythmicity and synaptic integration. They are primarily recognized for their role in pacemaking and thus contribute critically to the firing patterns of various neuronal types, including cortical and hippocampal neurons [29,30]. These channels enable subthreshold synaptic inputs to be filtered, thus shaping the temporal summation and resonance of neuronal firing [29]. The interplay of HCN channels with neurotransmitters such as serotonin further illustrates their potential role in modulating spike probability in neuronal axons, thus contributing to the intricate dynamics of neural signaling [31,32,33].

The peripheral nervous system (PNS) also utilizes HCN channels to modulate pain sensitivity and neuronal excitability. In specific contexts, such as inflammation-induced pain states, HCN channels have been implicated in enhancing neuronal responsiveness, thus affecting overall sensory processing. Notably, the peripheral application of HCN channel blockers offers a therapeutic avenue to mitigate side effects linked to the modulation of central or systemic HCN channels, suggesting their versatile roles depending on their location within the body [34].

HCN 1–4 channels are variably expressed throughout the central nervous system (CNS) and PNS. The expression of HCN-1 and HCN-2 channels is predominant, particularly in regions responsible for sensory signal transduction and pain modulation, while HCN-3 and HCN-4 exhibit more limited expression in these contexts. HCN-1 and HCN-2 channels are notably expressed in sensory neurons located within the dorsal root ganglion (DRG) and are implicated in pain transmission pathways. In contrast, although all four isoforms are acknowledged to be present, the expression patterns for HCN-3 and HCN-4 are notably less prevalent in the DRG and more concentrated in specific non-sensory structures. HCN-3 has been reported to have variable distributions in the CNS, with some studies suggesting its involvement in modulating certain intrinsic properties of neurons in the brain. HCN-4, while primarily recognized for its role in cardiac pacemaking, is also present in the brain but does not exhibit the same levels of expression associated with pain pathways such as HCN-1 and HCN-2 [31,34,35,36].

### 4.2. Postoperative Stress, Anxiety, and Pain

The role of ivabradine in managing pain, stress levels, and anxiety following surgical procedures, particularly cardiac surgeries, is an area of increasing investigation due to the drug’s unique pharmacological properties (Table 4). One of them is that it inhibits not only the HCN-4 but all its isoforms (1–4) with similar half maximal inhibitory concentration values (Table 3). Its effects on those aspects have been studied mainly in animal models and much less in humans. HCN channels are widely expressed throughout the heart as well as in both the peripheral and central nervous systems. Increases in hyperpolarization-activated cation currents, carried by HCN channels, have been implicated in the onset and maintenance of neuropathic pain; as a result, ivabradine, which could, even if not selectively, act on these channels, potentially reduces postoperative neuropathic and inflammatory pain, in addition to the other effects mentioned above. HCN-2 channels could play a role in inflammatory neuropathic pain responses, but there is no specific blocker of HCN-2 channels licensed for use in humans [9,31,37,38,39]. However, it should be noted that there are also studies in humans (with a very small sample of patients) that did not show an effect of the drug in reducing neuropathic pain [40].

After cardiac surgery, patients often experience heightened stress and anxiety due to physiological and psychological factors. The surgical experience itself, compounded by pain and alterations in physical health, can exacerbate stress hormone release, including epinephrine and norepinephrine. Research has demonstrated that ivabradine effectively mitigates these increases in HR and stress-induced hormone release. Specifically, pre-treatment with ivabradine in a laboratory setting resulted in lower stress-related elevations in epinephrine levels, indicating a modulatory effect on the neuroendocrine response to stress [10]. This suggests that ivabradine could play a valuable role in creating a calmer physiological state post-surgery, potentially alleviating anxiety in patients recovering from cardiac operations [11].

The integrative effects of ivabradine on both stress and pain management can notably impact patient rehabilitation processes. A study highlighted that lower HRs induced by ivabradine are correlated with improved overall quality of life indicators in patients experiencing chronic heart failure or undergoing surgery [41]. It is reasonable to extend these findings to cardiac surgery recovery, where maintaining a lower HR might assist in promoting emotional and psychological stability, thereby limiting anxiety responses precipitated by surgical trauma.

This dual action—reducing anxiety and ensuring pain relief—positions ivabradine as a multifaceted agent in the postoperative care of cardiac surgery patients. By balancing HR control with mental well-being, ivabradine may emerge as a standard therapeutic intervention in the postoperative phase, particularly for those at risk of high anxiety and stress. This evolving understanding highlights the distinct intersection of cardiology and psychiatry concerning postoperative care. Further clinical trials extending to humans will be essential in solidifying ivabradine’s role in this complex interplay of recovery, particularly as patient-centered care continues to gain precedence in treatment paradigms.

## 5. Conclusions and Future Directions

When targeting sinus tachycardia specifically, ivabradine has been observed to effectively reduce resting HRs in postoperative patients. For instance, in patients undergoing CABG, ivabradine treatment leads to significant HR control, thereby contributing to better myocardial performance through the increase in stroke volume. This achievement not only optimizes diastolic function but also serves to reverse adverse myocardial remodeling processes, thus enhancing the overall cardiovascular recovery trajectory. Furthermore, the mechanisms underlying ivabradine’s action, such as the inhibition of HCN channels, prevent excessive HR increases while preserving left ventricular contractility, making it a well-rounded pharmacological intervention for patients suffering from chronic heart failure and acute low cardiac output scenarios. Ivabradine represents a significant advancement in the pharmacological management of low cardiac output syndrome, especially in patients intolerant to other HR-reducing therapies.

A clinical question that arises is the desired extent of ivabradine-induced HR reduction from the baseline level and whether there is an ideal HR target in these patients. The question is difficult to answer, but the use of transthoracic echocardiography has been proposed to estimate the ideal target by assessing the overlap between the E- and A-waves, as well as the deceleration time. Avoiding the tachycardia-induced E- and A-wave overlapping using echocardiographic monitoring results in complete left ventricular relaxation and better ventricular filling volume. Izumida et al. proposed a formula using deceleration time to estimate the ideal HR that achieves a zero overlap between E-and A-waves in patients with heart failure, which could potentially be applied and studied in cardiac surgery patients, given that “reduction in HR” seems like a fairly general term and is not individualized at all [42,43].

In scenarios where postoperative atrial fibrillation is a concern, ivabradine demonstrates a remarkable profile that can attenuate the incidence of postoperative arrhythmias. Evidence suggests that ivabradine treatment leads to lower rates of surgery-induced AF compared to traditional beta-blockers, as well as their co-administration, providing a safe alternative or a valuable addition to traditional beta-blocker therapy for patients with compromised cardiac function. The potential benefits of ivabradine in reducing adverse cardiac events are further underscored by studies emphasizing its role in managing these arrhythmias effectively through precise HR control and minimization of adrenergic stress responses. On the contrary, an important result was obtained from the SHIFT trial which did not include cardiac surgery patients but rather patients with chronic heart failure who were randomly assigned to ivabradine and placebo treatment groups. Patients who received ivabradine had a higher incidence of AF than patients in the second group [306 (9%) vs. 251 (8%), *p* = 0.012] during a median follow-up of 22.9 months [44]. This result may indicate a different utility of the drug in chronic patients compared to patients with an acute disease.

Ivabradine’s anti-inflammatory and antioxidative properties in endothelial cells are mediated through two interrelated, yet distinct, mechanisms. First, there is direct intracellular modulation of signaling pathways, notably the mTOR/eNOS axis, which curtails oxidative stress and inflammatory activation under conditions of low shear stress. Second, there is an indirect biomechanical effect whereby ivabradine alters local blood flow dynamics, thereby modulating shear stress—a key regulator of endothelial phenotype and inflammatory response. These dual mechanisms propose that ivabradine might have therapeutic potential in vascular disorders where endothelial dysfunction and inflammation play central roles, thus supporting its broader application in cardiovascular medicine beyond its established use in HR management.

One of the critical challenges faced in postoperative care is the management of stress, anxiety, and pain, all of which can exacerbate cardiovascular strain and impede recovery. Studies have indicated that ivabradine can significantly lower postoperative stress-related elevations in stress hormones, such as epinephrine and norepinephrine, inducing a calmer neuroendocrine response in patients. This reduction in stress markers could create an environment favorable for healing, paralleling findings where lower HRs correlated with improved overall quality of life indicators in chronic heart failure patients undergoing surgeries. Moreover, ivabradine’s analgesic properties enhance its therapeutic profile, suggesting that it could provide comprehensive management of physical discomfort as well as emotional distress.

Despite the promising data, ongoing research is pivotal to fully elucidate ivabradine’s role and optimize its therapeutic applications within the perioperative context. As noted, the nuanced understanding of the drug’s dose–response relationships will further refine its integration into clinical protocols, especially to maximize its benefits while circumventing potential adverse outcomes associated with other standard treatments like beta-blockers, particularly in vulnerable patient populations. Overall, as our understanding deepens, ivabradine may establish itself as a therapy in the multimodal approach to improving cardiac surgery outcomes, particularly for patients vulnerable to tachyarrhythmias, impaired systolic and/or diastolic LV function, and heightened stress and pain responses. However, large randomized clinical trials are needed in order to confirm this approach. Moreover, these studies will clarify if the results of ivabradine are dependent on the specific type of cardiac surgery.

**Figure 1:**
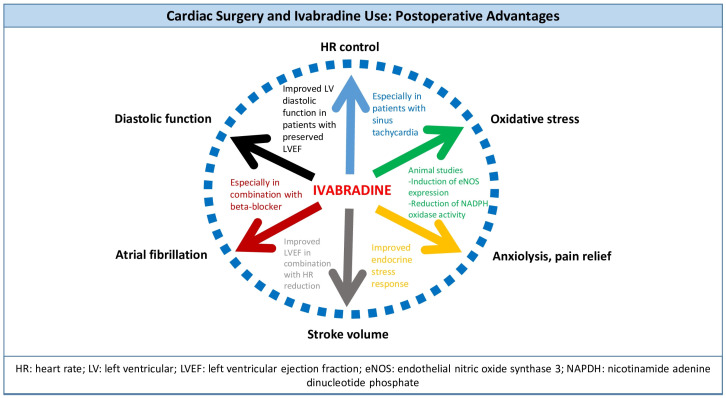
The multifaceted action of ivabradine in the context of perioperative management of patients undergoing cardiac surgery.

In summary, ivabradine presents a multifaceted therapeutic option in the management of key challenges in the postoperative period of cardiac surgeries (Figure 1). By effectively controlling HR, reducing arrhythmia burden, and lessening stress and pain levels, it contributes positively to the patient’s recovery trajectory. Collectively, these attributes highlight ivabradine’s potential to not only enhance cardiovascular stability but also promote overall well-being during the critical perioperative phase, paving the way for robust postoperative rehabilitation and improved patient outcomes.

## Figures and Tables

**Table 1:** Τhe effect of ivabradine on endothelial cell behavior, reducing inflammation and oxidative stress. The studies above are based on animal models. LSS: low shear stress, eNOS: endothelial nitric oxide synthase, mTOR: mammalian target of rapamycin, WSS: wall shear stress, VCAM-1: vascular cell adhesion protein 1, HR: heart rate, MCP-1: monocyte chemotactic protein-1, and NADPH: nicotinamide adenine dinucleotide phosphate.

Study (Year)	Key Question	Possible Pathophysiological Pathway	Main Results
Li et al. (2016) [18]	Vascular anti-inflammatory effects	Possible impact on LSS-induced inflammation and endothelial injury and the role of eNOS	-Endothelial protection from LSS-induced inflammation and oxidative stress -Slowdown in the progress of plaque formation by altering eNOS activity (mediated by the mTOR pathway)
Luong et al. (2016) [19]	Vascular anti-inflammatory effects	Possible vascular protection by increasing WSS to reduce vascular inflammation	-Reduction in expression of pro-inflammatory VCAM-1 -Enhancement in expression of anti-inflammatory eNOS at the inner curvature of the aorta -HR reduction and WSS enhancement in the aorta
Custodis et al. (2008) [20]	Effects on endothelial function and atherogenesis	Possible improvement in endothelial function and reduction in oxidative stress and atherosclerotic plaque formation through selective HR reduction	Selective HR reduction: -had no effect on the number of endothelial progenitor cells -did not alter aortic eNOS activity -decreased MCP-1 mRNA -decreased markers of superoxide production and lipid peroxidation in the aortic wall -reduced NADPH oxidase activity
Kröller-Schön et al. (2011) [21]	Effects on endothelial function	Possible influence of ivabradine on oxidative stress and endothelial dysfunction through HR reduction	-Antioxidative effects occurred through decreased NADPH oxidase activity and the prevention of eNOS uncoupling -Attenuation of angiotensin II signaling and additional vascular benefits

**Table 3:** Studies examining the role of ivabradine in POAF. POAF: postoperative atrial fibrillation, CABG: coronary artery bypass graft, and LV: left ventricular.

Study (Year)	Type of Study	Sample Size	Design of the Study	Clinical Effect	Possible Role in a Specific Perioperative Clinical Context
El-saied et al. 2024 [30]	Prospective interventional study	50 patients	Patients who underwent elective CABG surgery were divided into Group I, who received both ivabradine and bisoprolol, and Group II, who received bisoprolol only, 48 h before and one week after surgery	There was a statistically significant increase in the POAF incidence among patients in Group II (*p* = 0.01)	POAF
Abdel-Salam et al. 2016 [7]	Prospective, nonrandomized, multicenter, and observational study	740 patients	Patients scheduled for elective CABG operation were divided into three treatment groups: ivabradine and bisoprolol group, ivabradine-only group, and bisoprolol-only group (each treatment started 48 h before surgery and ended one week afterwards)	The incidence of POAF was significantly lower in the ivabradine and bisoprolol group 3 compared to the ivabradine-only group and bisoprolol-only group (*p* < 0.001 for both)	POAF
Iliuta et al. 2014 [8]	Open-label randomized clinical trial	527 patients	Patients with conduction abnormalities or LV systolic dysfunction underwent CABG or valvular surgery and were randomized into three treatment groups: the ivabradine-only group, the metoprolol-only group, and the metoprolol plus ivabradine group (each treatment started 2 days before surgery and ended ≥10 days postoperatively)	In-hospital POAF occurred less frequently with combined therapy than with metoprolol or ivabradine alone (*p* < 0.001)	POAF

**Table 4:** Investigation of the possible role of ivabradine in the context of perioperative management of stress, pain, and anxiety during cardiac surgery. NP: neuropathic pain, HCN: hyperpolarization-activated cyclic nucleotide-gated, MAP: mean arterial pressure, HR: heart rate, BP: blood pressure, and -: no correlation was shown.

Study (Year)	Type of Study	Aim of the Study	Key Pathophysiological Mechanism	Main Results	Possible Role in the Context of Perioperative Management of Stress, Pain, and Anxiety
Noh et al. (2014) [37]	Animal study	Investigation of the effects of ivabradine on NP	Peripheral nerve injury increases the excitability of primary sensory neurons, triggering the onset of NP. Changes in HCN channels are implicated in this process	-Ivabradine significantly reduced NP (mechanical allodynia) -MAP was maintained -A HR reduction of 15% was observed	Pain management
Tanaka et al. (2022) [38]	Randomized double-blinded, placebo-controlled, and crossover study	Evaluation of the analgesic effect of 2-day administration of ivabradine on a capsaicin pain model	Ivabradine alleviates mechanical allodynia with NP via inhibition of the HCN current in large dorsal root ganglion	-There was no significant difference in spontaneous pain or flare size between the ivabradine and placebo groups -The area of dynamic mechanical allodynia in the ivabradine group was significantly smaller	Pain management
Ohashi eta al. (2022) [39]	Animal study	Investigation of the spinal action and cellular mechanisms underlying ivabradine’s analgesic effects against inflammatory pain	Spinal responses mediated by HCN channels on primary afferent terminals are affected by the central and peripheral administration of ivabradine	-Spinal responses mediated by HCN channels on primary afferent terminals are suppressed by the administration of ivabradine -Ivabradine preferentially acts on C-fiber terminals of spinal dorsal horn neurons, inducing a stronger inhibition of neuronal excitability in inflammatory pain	Pain management
Miyake et al. (2019) [9]	Animal study	Evaluation of ivabradine’s effect on inflammatory pain	Ivabradine acts on peripheral sensory neurons and has inhibitory effects on neuropathic pain. It also acts on HCN channels, which are involved in the modulation of inflammatory pain	-Ivabradine affects inflammation responses, including the accumulation of leukocytes and TNF-alpha expression -Ivabradine also plays a role in neuropathic pain reduction	Pain management
Lee et al. (2019) [40]	Single-center, randomized, double-blind, placebo-controlled, and 2-period crossover trial	Evaluation of the influence of ivabradine on capsaicin-induced hyperalgesia and pain in healthy human subjects	Possible analgesic potential of peripherally acting non-selective HCN blockers	-Ivabradine caused a HR reduction -There were no significant effects of ivabradine on the primary outcome, defined as a difference in the effects of placebo and ivabradine on the area of punctate hyperalgesia	
Ondicova et al. (2019) [10]	Animal study	Investigation of ivabradine’s stress-induced effects on HR, BP, and neuroendocrine stress response	Possible influence of ivabradine on signaling that accompanies a stress-induced rise in HR and the extent of the neuroendocrine stress response (sympathoadrenal system and hypothalamic–pituitary–adrenocortical axis)	-Ivabradine significantly lowers values of HR and BP -It significantly reduces handling-induced epinephrine and norepinephrine release into the bloodstream	Stress reduction
Woodman et al. (2023) [11]	Animal study	Evaluation of the effect of ivabradine treatment on anxiety reduction	A reduction in funny channel currents brought about by the targeted disruption of HCN gene expression and the inhibition of cyclic nucleotide binding to HCN channels in the brain has been shown to improve coping in animal models of stress	-Ivabradine reduces resting HR -Stressed mice treated with ivabradine displayed significantly greater exploratory behavior according to the qualitative cognition and anxiety assessment	Anxiety management and stress reduction

## Data Availability

No new data were created in this study.

## Abbreviations

The following abbreviations are used in this manuscript:

HR	heart rate
HCN	hyperpolarization-activated cyclic nucleotide-gated
cAMP	cyclic adenosine monophosphate
AF	atrial fibrillation
CABG	coronary artery bypass grafting
eNOS	endothelial nitric oxide synthase
mTOR	mammalian target of rapamycin
VCAM-1	cytokine vascular cell adhesion molecule 1
MCP-1	monocyte chemotactic protein-1
NADP	nicotinamide adenine dinucleotide phosphate
ROS	reactive oxygen species
LV	left ventricle
LVEF	left ventricular ejection fraction
NYHA	New York Heart Association
LCOS	low cardiac output syndrome
POAF	postoperative atrial fibrillation
ICU	intensive care unit
PNS	peripheral nervous system
CNS	central nervous system
DRG	dorsal root ganglion
